# Outcomes of the Surgical Management of Atrial Isomerism and Functional Single Ventricle: A Single-Centered Cohort From China

**DOI:** 10.3389/fcvm.2021.664752

**Published:** 2021-09-22

**Authors:** Ming-Hui Zou, Fan Cao, Li Ma, Wei-Dan Chen, Wen-Lei Li, Jia Li, Xin-Xin Chen

**Affiliations:** ^1^Department of Cardiovascular Surgery, Guangzhou Women and Children's Medical Center, Guangzhou Medical University, Guangdong, China; ^2^Guangdong Provincial Key Laboratory of Research in Structural Birth Defect Disease, Department of Pediatric Surgery, Guangzhou Women and Children's Medical Center, Guangzhou Medical University, Guangdong, China

**Keywords:** right atrial isomerism, left atrial isomerism, single ventricle, Fontan, Heterotaxy syndrome

## Abstract

**Objectives:** The management of atrial isomerism with complex congenital heart disease remains challenging. Experience has been largely obtained in advanced countries. The clinical diversity is greater in China. We evaluated the early- and medium-term outcomes of surgical treatment of these patients.

**Methods:** We reviewed 86 patients of atrial isomerism with complex congenital heart disease undergoing varied surgeries in our center in 2008–2020. Cox regression models were used to analyze the risk factors for mortality.

**Results:** There were 75 cases of right and 11 of left atrial isomerism. Eighty-three (96.5%) patients underwent single-ventricle staged palliation approach, with 10 early and 7 late deaths. The overall 1-, 5-, and 10-year survival rates were 84.7, 79.3, and 79.3%, respectively. Thirty-six (43.4%) patients completed the Fontan procedure with median age of 48 months and freedom from death or Fontan failure at 1-, 5-, and 8-years were 94.4, 87.4, and 80.7%, respectively. Concomitant total anomalous pulmonary venous connection [hazard ratio (HR): 5.15 (1.95–12.94), *p* = 0.008], more than moderate atrioventricular valve regurgitation [HR: 4.82 (2.42–6.79), *p* = 0.003], and the need for first-stage palliative surgery [HR: 4.58 (1.64–10.76), *p* = 0.015] were independent risk factors for mortality.

**Conclusions:** Despite even greater clinical diversity, the surgical outcomes of atrial isomerism with complex congenital heart disease are improving in China. The early and intermediate outcomes are comparable to many previous reports. Concomitant total anomalous pulmonary venous connection, moderate or severe atrioventricular valve regurgitation, and the need for a first-stage palliative surgery are still independent risk factors for mortality.

## Introduction

Atrial isomerism is a condition in which the thoracoabdominal organs present abnormal lateral arrangement and is frequently associated with complex cardiovascular anomalies. Patients with isomerism can be stratified into the subsets of asplenia syndrome and polysplenia syndrome or the subsets of isomerism of the right atrial appendages [right atrial isomerism (RAI)] and isomerism of the left atrial appendages [left atrial isomerism (LAI)] (1). Cardiovascular mortality in early childhood is estimated at about 10% or more, even in the current era (2, 3). Fortunately, recent reports from developed countries showed that the survival of atrial isomerism with cardiac malformation is continuing to improve, and most patients can survive into adulthood (4–8).

However, in China and other developing countries, there is substantial heterogeneity in the age of patients at the first visit and consequent questionable potential for long-term survival. We previously reported our early- and middle-term surgical outcomes in patients with atrial isomerism (9, 10). As cases accumulated and follow-up time increased, the late outcomes and risk factors were better documented. Therefore, this present study was undertaken to review our up-to-date single institutional experience in the past 13-years.

## Patients and Methods

Following the approval by the Institutional Ethics Board, we performed a retrospective chart review of patients diagnosed as atrial isomerism at Guangzhou Women and Children's Medical Center from January 2008 to December 2020. The diagnostic criteria were referred to the previous definition (1). Patients with situs inversus totalis or without thoracoabdominal laterality defect were excluded.

### Data Collection

The demographic characteristics, medical record, echocardiographic and angiographic data, morphological feature, and operative information for all patients were reviewed. Information regarding their clinical status was obtained from the medical records and by contacting the referring cardiologist.

### Morphological Features

Bronchial, atrial appendage, and abdominal morphology and/or situs inversus totalis were determined based on echocardiography, computed tomography (CT), and operative reports. Abdominal ultrasound was used to assess splenic anatomy. According to the atrial morphology, bronchial-pulmonary morphology, and asplenia or polysplenia, all patients were stratified into the LAI and RAI groups. Anatomic characteristics were described based on the sequential segmental analysis created by von Praagh. Extracardiac total anomalous pulmonary venous connection (TAPVC) included supracardiac, infracardiac, and mixed type. Atrioventricular (AV) connections included biventricular and univentricular atrioventricular connection. The atrioventricular valve (AVV) morphology was recorded as common, single mitral valve, single tricuspid valve, or both mitral and tricuspid valve. The degree of AVV regurgitation is judged qualitatively from the color Doppler signal because of a lack of accurate quantitative methods for assessing AVV regurgitation in those with single-ventricle anatomy. In our center, the degree of AVV regurgitation was graded as absent, mild, moderate, or severe (11). When the dominant ventricle morphology was difficult to identify, it was recorded as ambiguous. The discordant ventriculoarterial connection was defined as the aorta arising from the right ventricle, including transposition and double-outlet right ventricle (DORV).

### Operative Strategies

In our center, the surgical strategies were based on a multidisciplinary assessment. The decision as to whether a single-ventricle pathway will have to be pursued is usually dependent on whether complex venous connections, either systemic or pulmonary, can be baffled and septated within a common atrium or, more commonly, whether the patient with DORV and complete atrioventricular canal (CAVC) can be given a biventricular repair through complex intraventricular baffling of the left ventricle to the right-sided aorta.

The first palliative surgery included a systemic-to-pulmonary shunt, pulmonary artery banding (PAB), repair of TAPVC or AVV regurgitation, and relief of left ventricle outflow tract. As recommended by heart centers in most developed countries, the timing of the first palliative surgery was the neonatal period or within 3 months of age. The second stage of bidirectional Glenn shunt was recommended at 4–6 months, and the third stage of Fontan procedure was recommended at 3–4-years old.

Our surgical indication for AVV regurgitation was moderate or more. The timing of repair for moderate AVV regurgitation was concomitant with the bidirectional Glenn shunt or Fontan procedure. But for those with severe AVV regurgitation, the AVV repair or replacement was completely separate to avoid concurrent Glenn or Fontan procedure.

Patients who were judged suitable for Fontan procedure underwent further cardiac catheterization and angiography to determine the anatomical features of the pulmonary arteries, aortopulmonary collateral arteries, and systolic and diastolic ventricular function. The mean pulmonary artery pressure (mPAP) and pulmonary vascular resistance (PVR) were noted from catheterization records.

### Follow-Up and Reintervention

All patients were followed up regularly at 1, 3, 6 months, and 1-year postoperatively, and once per year thereafter. Re-examination by echocardiography, electrocardiography, and chest X-ray was performed, and cardiac CT and catheterization were performed as needed. The follow-up focused on blood oxygen saturation, atrioventricular valve regurgitation, cardiac function, and quality of daily life, and surgical or transcatheter reintervention was performed when needed.

### Statistical Analysis

The primary outcomes included early and late death, reintervention, Fontan failure, and survival rate. Early death was defined as any death occurring within 30 days after surgery or mortality before hospital discharge. Fontan failure was defined as any of the following events: heart transplantation, Fontan takedown or conversion, protein-losing enteropathy (PLE), plastic bronchitis, or New York Heart Association (NYHA) functional class III or IV at follow-up. Major perioperative complications included low cardiac output syndrome (LCOS), multiple organ dysfunction syndrome (MODS), prolonged pleural effusion (>14 days), severe arrhythmias, bleeding, and thromboembolic events.

Data were expressed as mean ± SD, median (range), or frequency (%) when appropriate. Between-group differences were compared using a *t*-test or chi-square test when appropriate. The Kaplan–Meier method was used to estimate survival time, and the log-rank test was used to compare differences in survival rates. Univariate Cox regression analysis was performed, and variables with a *p* < 0.1 were included. A forward stepwise regression method was used based on maximum likelihood estimation. A *p* < 0.05 was considered significant. SPSS 22.0 (IBM, Armonk, NY) was used for statistical analyses.

## Results

### Patient Characteristics

During the study period, 86 (60 male, 26 female) consecutive patients with atrial isomerism and complex congenital heart disease underwent surgical intervention. Seventy-five (87.2%) patients were identified to have the RAI leaving only 11 patients in the LAI group. The median age of RAI and LAI group were 11.3 months (range: 6 days to 150 months) and 14.5 months (range: 2–96 months), respectively. Thirty-seven (49.3%) patients showed asplenia in RAI group, and six (54.5%) showed polysplenia in the LAI group. Patient demographic and morphologic variables are presented in Table 1.

**Table 1:** Demographic and anatomic characteristics of patients with atrial isomerism (*n* = 86).

**Variables**	**RAI (*****n*** **= 75)**	**LAI (*****n*** **= 11)**
Gender (M/F)	54/21	6/5
Age (months) (median, range)	11.3 (0.2–150)	14.5 (2–96)
Weight (kg) (median, range)	7 (3.0–36.0)	10 (4.0–21.0)
**Spleen**		
Asplenia	37 (49.3%)	0 (0.0%)
Polysplenia	1 (1.3%)	6 (54.5%)
Normal	37 (49.4%)	5 (45.5%)
**Cardiac position**		
Levoverted	17 (22.7%)	1 (9.1%)
Levocardia	32 (42.6%)	5 (45.4%)
Dextroverted	22 (29.3%)	4 (36.4%)
Dextrocardia	4 (5.3%)	1 (9.1%)
Apicocaval juxtaposition	20 (26.7%)	2 (18.2%)
**Systemic veins connection**		
Left SVC	10 (13.3%)	0 (0.0%)
Right SVC	22 (29.3%)	2 (18.2%)
Bilateral SVC	43 (57.4%)	9 (81.8%)
Interrupted IVC-azygous continuation	0 (0.0%)	9 (81.8%)
Interrupted IVC-hemizygous continuation	0 (0.0%)	1 (9.1%)
Separate IVC-hepatic vein orifice	6 (8.0%)	0 (0.0%)
Normal IVC present	69 (92.0%)	1 (9.1%)
**Pulmonary venous connection**		
PAPVC	–	3 (27.3%)
TAPVC	50 (66.7%)	2 (18.2%)
Extracardiac TAPVC	26 (34.7%)	1 (9.1%)
Intracardiac TAPVC	49 (65.3%)	1 (9.1%)
Obstructed TAPVC	17 (22.7%)	2 (18.2%)
Common atrium	45 (60.0%)	6 (54.5%)
**Atrioventricular connection**		
Univentricular	53 (70.7)	6 (54.5%)
Biventricular	22 (29.3)	5 (45.5%)
**AVV morphology**		
Common AVV	57 (76.0%)	5 (45.5%)
Mitral valve	10 (13.3%)	0 (0.0%)
Tricuspid valve	3 (4.0%)	4 (36.4%)
Mitral and tricuspid valve	5 (6.7%)	2 (18.1%)
**AVV regurgitation (≥moderate)**		
Before the first palliation	3 (14.3%)	3 (27.3%)
Before Glenn	21 (30.9%)	1 (1.5%)
Before Fontan	8 (22.2%)	0 (0.0%)
**Dominant ventricular morphology**		
Left	14 (18.7%)	1 (9.1%)
Right	33 (44.0%)	7 (60.6%)
Ambiguous	28 (37.3%)	3 (27.3%)
**Ventriculoarterial connection**		
Concordant (aorta from LV)	13 (17.3%)	3 (27.3%)
Discordant (aorta from RV)	62 (82.7%)	8 (72.7%)
**Pulmonary stenosis—valvular/subvalvular**	51 (68.0%)	7 (63.6%)
**Pulmonary atresia**	20 (26.7%)	1 (9.1%)
**Pulmonary hypertension**	4 (5.3%)	3 (27.3%)
**Coronary artery anomaly**	21 (28.0%)	4 (36.4%)
**Coarctation of the aorta**	0 (0%)	1 (9.1%)

*RAI, right atrial isomerism; LAI, left atrial isomerism; SVC, superior vena cava; IVC, inferior vena cava; PAPVC, partial anomalous pulmonary venous connection; TAPVC, total anomalous pulmonary venous connection; AVV, atrioventricular valve; LV, left ventricle; RV, right ventricle*.

### Surgical Algorithm

**Figure 1:**
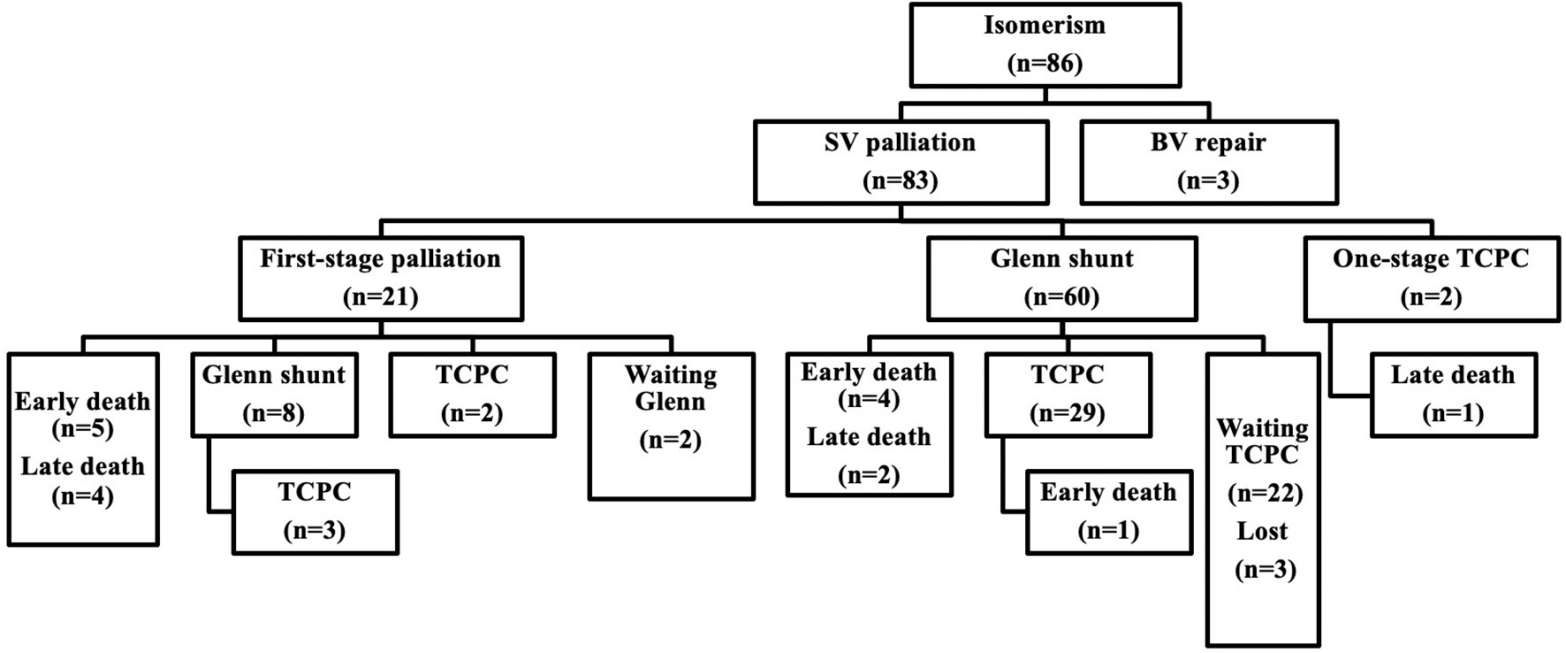
Flow chart of interventions in 86 patients with atrial isomerism.

Eighty-three (96.5%) patients underwent single-ventricle staged palliative surgery, and only three (3.5%) patients underwent biventricular repair pathway. A flow chart of surgical intervention is shown in Figure 1.

Twenty-one patients (25.3%, 21/83) required first-stage palliative surgery at a median age of 4.9 months (range: 0.2–95.4 months, including four neonates) and median weight of 4.5 kg (range: 3.0–21.5 kg). The first-stage palliative surgery details included modified Blalock–Taussig (B-T) shunt in 13 patients (14 times), TAPVC repair in 8, PAB in 7, central pulmonary artery reconstruction and pulmonary angioplasty in 2, AVV repair in 4, AVV replacement in 1, and atrial septal defect (ASD) enlargement in 1 patient.

Sixty-eight patients (81.9%, 68/83) underwent a bidirectional Glenn shunt at a median age of 14.3 months (range: 5.0–150.0 months) and preoperative SpO_2_ of 58 ± 18%. Glenn shunt included unilateral and bilateral procedures in 28 and 40 patients, respectively. Concomitant surgery included 20 patients of TAPVC repair, 18 of AVV repair, 17 of patent ductus arterious (PDA) ligation, 8 of pulmonary arterioplasty, 4 of PAB, 3 of AVV replacement, 3 of ASD enlargement, and 2 cases of cor triatriatum repair. Overall, thirty-six (52.9%, 36/67) cases were completed under cardiopulmonary bypass.

Thirty-six (43.4%, 36/83) patients finally completed Fontan procedure, including 4 patients of one-stage total cavopulmonary connection (TCPC) and 32 of multi-staged TCPC. The median age at Fontan completion was 48.0 months (range: 26.6–161.3 months), and the median weight was 14.9 kg (range: 7.3–27.0 kg). The mean interval between Fontan and Glenn shunt was 31 ± 13 months. Preoperative SpO_2_ was 72 ± 13%. Before Fontan, 26 (72.2%, 26/36) patients underwent cardiac catheterization with a mPAP of 13.7 ± 2.8 mmHg and PVR of 2.9 ± 1.3 Wood units. Extracardiac conduits Fontan was used in 33 patients with a diameter of 18–22 mm and intra-atrial tunnel Fontan in 3 patients. Thirty-one (86.1%, 31/36) cases had a 3-mm fenestration. Concomitant surgery included 6 cases of AVV repair, 6 of hepatic vein-extracardiac conduit end-to-side anastomosis, 2 of pulmonary arterioplasty, and 1 of pulmonary vein restenosis repair. Ten (27.8%, 10/36) patients underwent the Fontan surgery with cardiopulmonary bypass and cardioplegia.

### Early Mortality and Morbidity

Ten (12.0%, 10/83) patients who underwent single-ventricle palliative pathway suffered from early mortality, which included 5 (23.8%) in first-stage palliative surgery, 4 (5.9%) in Glenn, and 1 (2.8%) in Fontan procedure. The major causes of death included LCOS in 6 patients, residual pulmonary venous stenosis in 2, severe capillary leakage in 1, and multiple infection and MODS in 1. There was no early death in the three patients who underwent biventricular repair. The details of all death cases are listed in Table 2.

**Table 2:** The details of all death cases (*n* = 17).

**No**	**Gender**	**Age**	**Diagnosis**	**Surgical method**	**Death type**	**Cause of death**
1	F	8-years	LAI/CAVVR (severe)/heart failure	CAVV replacement	Early death	LCOS
2	M	12 days	RAI/TAPVC (Infra)	TAPVC repair + mBTS	Early death	LCOS
3	M	6 days	RAI/TAPVC (mixed)	TAPVC repair + mBTS	Early death	Capillary leakage syndrome, renal failure, MODS
4	M	45 days	RAI/ob-TAPVC (mixed)/discontinuous central PA	PA reconstruction + mBTS + TAPVC repair	Early death	Residual PVS
5	M	40 days	RAI/ob-TAPVC (Infra)/PAH	TAPVC repair + PAB	Early death	hypoxemia, LCOS
6	M	5.8-years	RAI/BSVC/TAPVC (supra)/CAVVR (moderate)	Bilateral Glenn shunt + TAPVC repair + CAVV repair	Early death	LCOS
7	M	14 months	RAI/BSVC/TAPVC (Intra)/CAVVR (severe)	Bilateral Glenn shunt + CAVV replacement	Early death	Multiple infection
8	M	6 months	RAI/TAPVC (Supra)	Glenn shunt + TAPVC repair	Early death	PVS, pulmonary hemorrhage
9	F	6.5-years	RAI/BSVC/TAPVC (Supra)	Bilateral Glenn shunt + TAPVC repair	Early death	LCOS
10	M	4.8-years	RAI/TAPVC (Supra)	The second stage TCPC	Early death	Hypoxemia, LCOS
11	M	2.6-years	LAI/CAVVR (moderate)/PAH	PAB	Late death	CAVVR (severe), death after the valve replacement
12	M	12 days	RAI/TAPVC (Supra)/ discontinuous central PA	PA reconstruction + mBTS + TAPVC repair	Late death	PVS
13	M	55 days	LAI/TAPVC (Intra)	mBTS	Late death	unclear
14	F	2-years	RAI/ob-TAPVC (Supra)	mBTS + TAPVC repair	Late death	unclear
15	M	1-year	RAI/ob-TAPVC (Supra)	Glenn shunt + TAPVC repair	Late death	Recurrent PVS, CAVVR (severe), heart failure
16	M	5 months	RAI/BSVC/TAPVC (Intra)	Bilateral Glenn shunt	Late death	Intestinal obstruction
17	M	4.4-years	RAI/TAPVC (Supra)	The first stage TCPC	Late death	PLE

*CAVVR, common atrioventricular valve regurgitation; CAVV, common atrioventricular valve; LCOS, low cardiac output syndrome; TAPVC, total anomalous pulmonary venous connection; mBTS, modified Blalock–Taussig shunt; MODS, multiple organ dysfunction syndrome; ob-, obstructed; PA, pulmonary artery; PVS, pulmonary venous stenosis; PAH, pulmonary arterial hypertension; PAB, pulmonary artery banding; BSVC, bilateral superior vena cava; TCPC, total cavo-pulmonary connection; PLE, protein-losing enteropathy*.

Other major perioperative complications included prolonged pleural effusion in 18 patients (including chylothorax in 6), transient arrhythmias in 12, diaphragmatic paralysis in 4, bleeding event in 3, delayed closure of the sternum in 3, nervous system thromboembolic event in 1, and extracorporeal membrane oxygenation (ECMO) support in one patient due to pulmonary hemorrhage and severe hypoxemia. The mean SpO_2_ at discharge after Glenn and Fontan procedure reached 84 ± 10 and 92 ± 8%, respectively. Ten (27.8%, 10/36) patients after Fontan suffered from prolonged pleural effusion.

### Follow-Up, Reintervention, and Intermediate Outcomes

**Figure 2:**
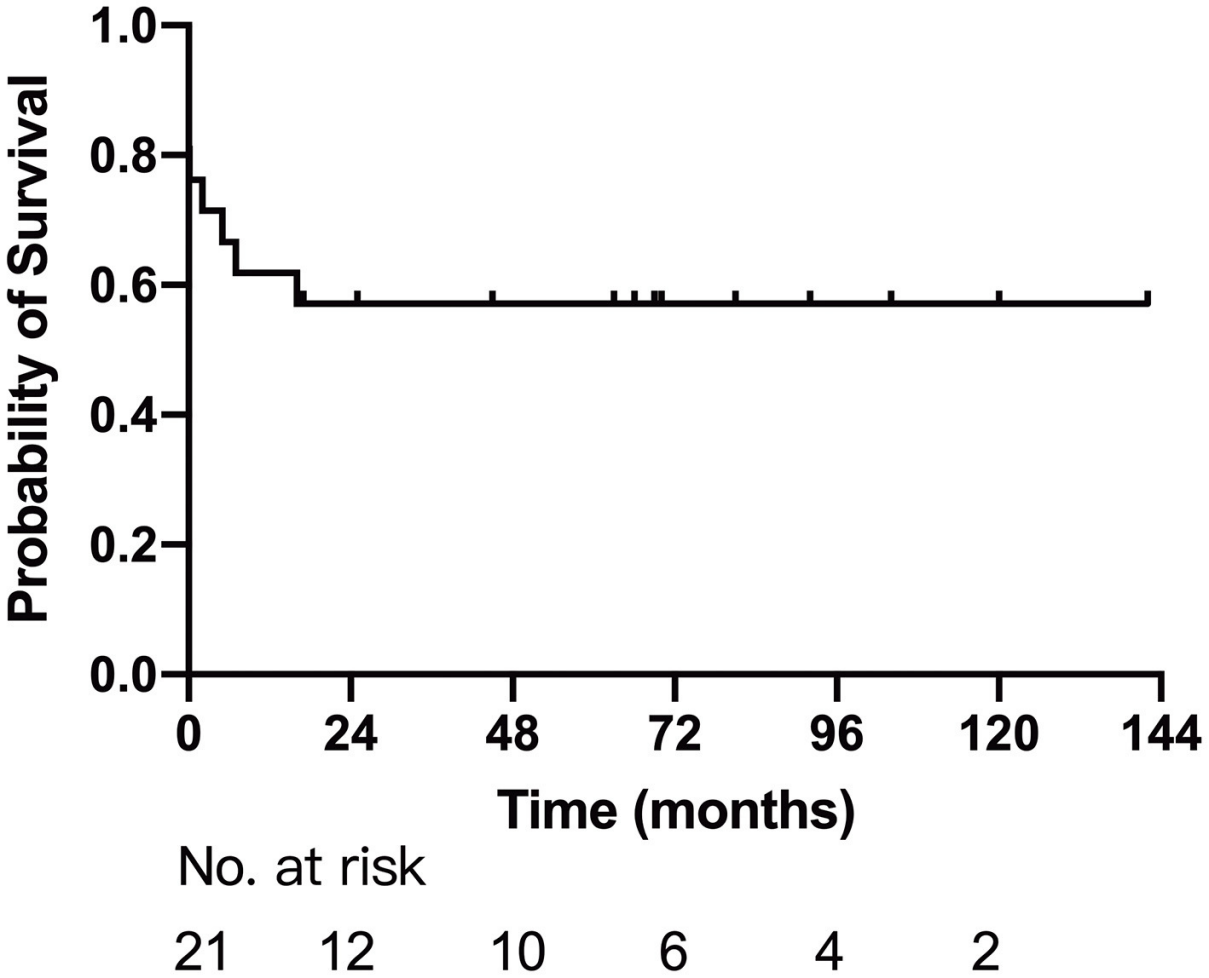
Survival for patients after first-stage palliative surgery.

Sixteen patients survived after first-stage palliative surgery, with a median follow-up of 33.5 months (range: 2–119 months). There were four late deaths; three of them were after modified B-T shunt, two of whom accepted concomitant TAPVC repair, developed pulmonary venous stenosis, and died 5 and 7 months after the surgery, respectively, and one had sudden death with unknown cause. The remaining one death was due to severe common AVV regurgitation and died after replacement of the common AVV. The details are described in Table 2. Eight patients successfully completed Glenn shunt, two directly underwent TCPC, and the remaining two were awaiting next-stage surgery. One and 5-year Kaplan–Meier survivals after first-stage palliative surgery were 61.9% (95% CI: 41.1–82.7%) and 57.1% (95% CI: 35.9–78.3%), respectively (Figure 2).

There were two late deaths after Glenn during a median follow-up of 72 months (range: 1–156 months). One of them was a 1-year-old male diagnosed with RAI with supracardiac type obstructed TAPVC and died 11 months after bidirectional Glenn shunt + concomitant TAPVC repair due to recurrent pulmonary vein obstruction and severe common AVV regurgitation and heart failure. The other death case was a 21-month-old boy who died of intestinal obstruction. He had underwent bilateral Glenn shunt when he was 5 months old.

**Figure 3:**
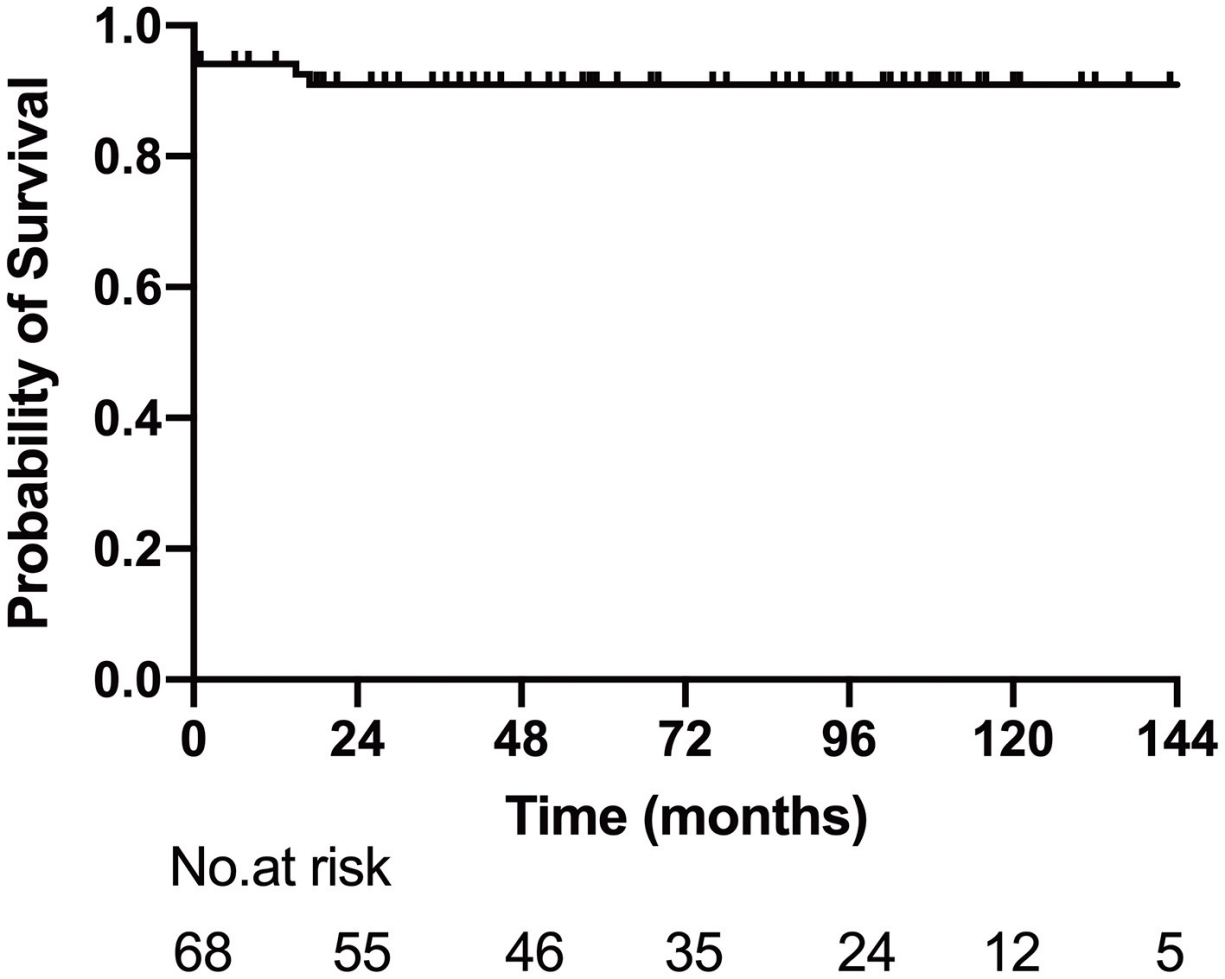
Survival for patients after Glenn shunt.

Reintervention was performed in six patients, including AVV replacement in four, AVV repair in one, and AVV replacement + pulmonary vein stenosis repair in one. The median interval between the Glenn procedure and reintervention was 15 months (range: 11–72 months). At the latest follow-up, 32 patients (47.1%, 32/68) completed Fontan procedure, 22 (32.4%, 22/68) were awaiting next-stage surgery, and 3 were lost to follow-up. The 1-, 5-, and 10-year survival rates after Glenn shunt were 94.1% (95% CI: 88.4–99.8%), 86.9% (95% CI: 84.1–97.8%) and 86.9% (95% CI: 84.1–97.8%) (Figure 3).

At a median follow-up of 67 months (range: 3–106 months) after Fontan procedure, there was one (2.8%) late mortality due to PLE 1-year after one-stage TCPC. Reintervention after Fontan was also performed in one patient. The patient had accepted a common AVV repair concomitant with the Glenn procedure. Echocardiography showed recurrence of severe AVV regurgitation 18 months after the surgery. Atrioventricular valvuloplasty was redone, and the Fontan procedure was successfully completed 21 months after the valve reoperation. However, at re-examination, echocardiography revealed severe recurrent AVV regurgitation again. Finally, the patient had to accept a fourth AVV replacement 12 months after Fontan and recovered well after the operation.

**Figure 4:**
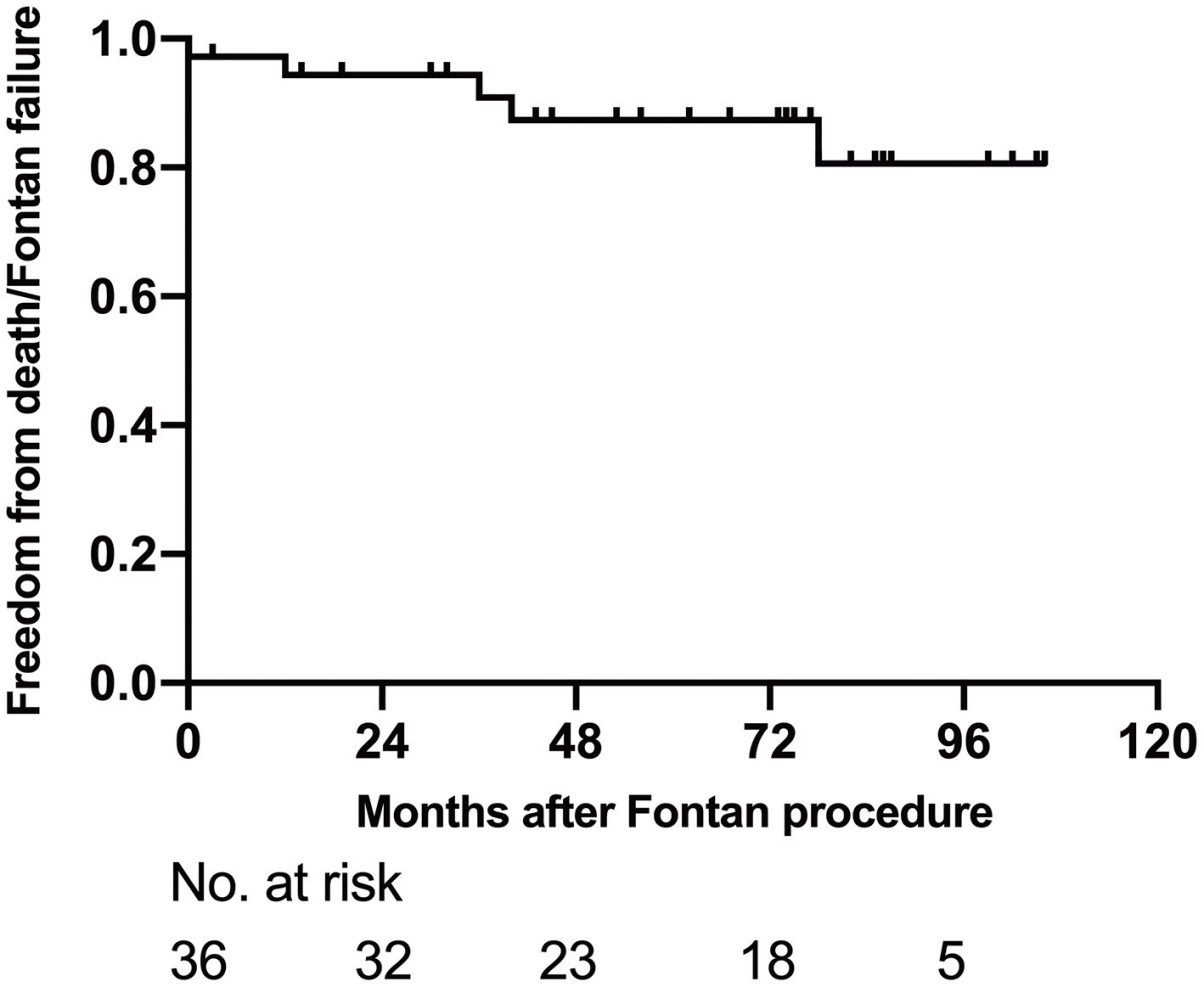
Survival for patients after Fontan completion.

At the latest follow-up, 31 (86.1%, 31/36) showed mild exercise intolerance with normal weight gain. However, three patients were diagnosed as Fontan failure and waiting for heart transplantation; two of them showed PLE, whereas one showed severely reduced ventricular systolic function. Other late complications after Fontan included supraventricular arrhythmia in six patients, moderate AVV regurgitation in three, and inferior vena cava thrombosis in one patient. The freedom from death or Fontan failure at 1-, 5-, and 8-years were 94.4% (95% CI: 86.8–100%), 87.4% (95% CI: 75.6–99.2%) and 80.7% (95% CI: 64.0–97.4%), respectively (Figure 4).

Besides, close follow-up was also conducted on the three patients with biventricular repair for 3–8-years. There was no late death with SpO_2_ above 95%, and their clinical status were good without exercise intolerance.

**Figure 5:**
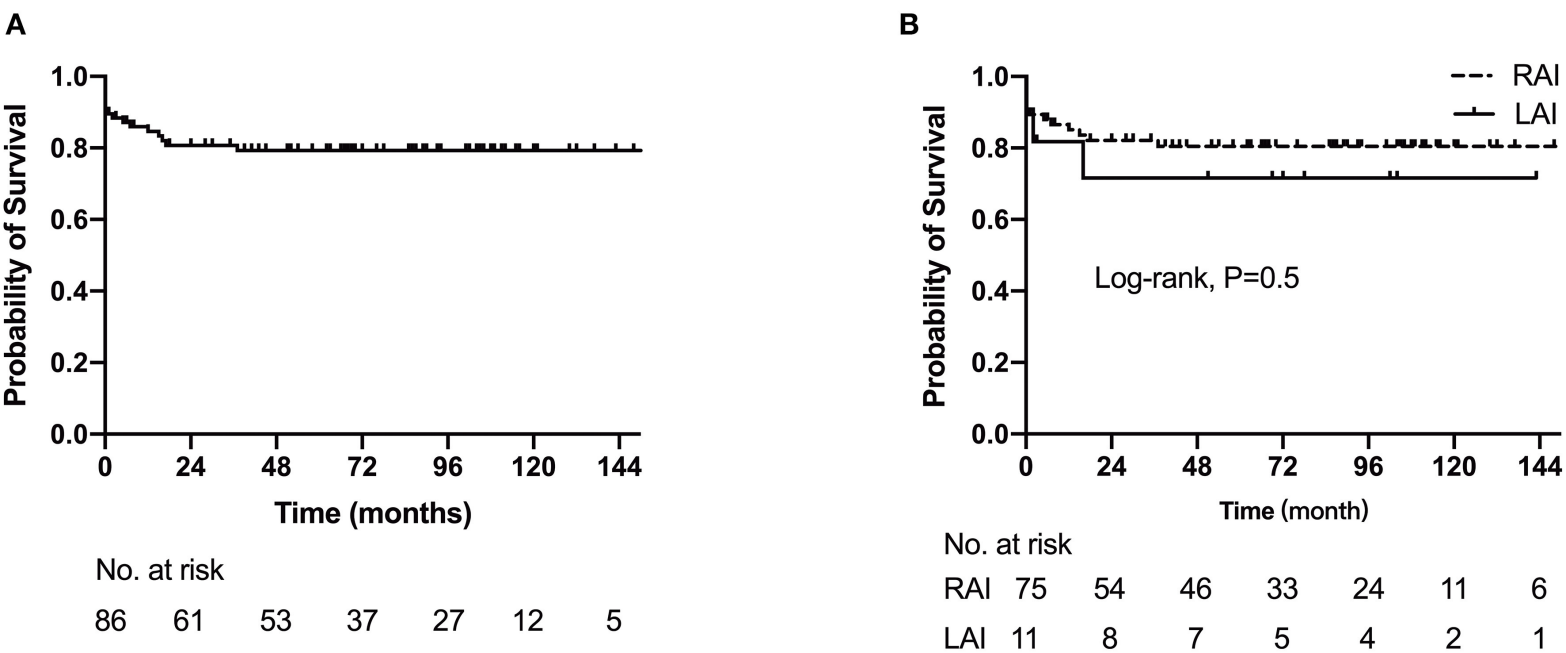
The Kaplan–Meier curves depict estimated survival. **(A)** Survival for the entire cohort of 86 patients. **(B)** Comparison of right and left atrial isomerism survival probability.

In the entire cohort, the 1-, 5-, and 10-year survival rates were 84.7% (95%CI: 77.1–92.3%), 79.3% (95%CI: 70.5–88.1%), and 79.3% (95%CI: 70.5–88.1%), respectively (Figure 5A). The 1- and 5-year survival rates of RAI were 85.1% (95% CI: 77.1–93.1%) and 80.5% (95% CI: 71.3–89.7%), respectively, while 1- and 5-year survival rates of LAI were both 80.0% (95% CI: 44.2–99.0%). There was no significant difference between the two groups (log rank, p = 0.509) (Figure 5B). Multivariate regression analysis showed extracardiac TAPVC [hazard ratio (HR): 5.15, 95% CI: 1.95–12.94, p = 0.008], more than moderate AVV regurgitation (HR: 4.82, 95% CI: 2.42–6.79, p = 0.003), and need for first-stage palliative surgery (HR: 4.58, 95% CI: 1.64–10.76, p = 0.015) were risk factors for mortality.

## Discussion

This study reviewed our experience in the management of 86 patients with atrial isomerism in our center over the past 13-years. Our data showed that despite even greater clinical diversity, the intermediate outcomes were improving, and 10-year survival rate reached 79.3%, which is comparable to many of the previous reports (5, 6). Consistent with previous studies, we found that extracardiac TAPVC, more than moderate AVV regurgitation, and requirement for first-stage palliative surgery were independent risk factors for mortality.

In surgical series, LAI is more common than RAI; in one surgical experience with 41 patients, 23 (56.1%) had LAI and 18 (43.9%) had RAI (12). However, several studies performed in Asian populations also showed a strong predilection (80% of cases) for RAI, suggesting there may be racial differences in the expression of LAI and RAI (13, 14). In the present study, up to 87% patients were identified as having RAI. Surprisingly, we have not encountered children with LAI in the past 5-years. Furthermore, most children underwent their first surgical intervention at an older age, which maybe had consequential adverse effect on the outcomes. This substantial heterogeneity in the age of patients at the first visit reflects the medical reality in China and many other developing countries and poses a huge challenge. Besides, although prenatal diagnosis has been popularized in China for several years, many newborns with complex heart defects are still born every year in remote areas.

Patients with LAI generally have relatively mild intracardiac malformations and sometimes can undergo biventricular repair. However, right isomerism often had more complex congenital heart defects, such as extracardiac obstructed TAPVC, unbalanced CAVC ventricle, univentricular atrioventricular connection, and discordant ventricular-arterial junction. Most of these patients had to accept a single-ventricle multi-staged palliative pathway. In this cohort, only three patients with LAI underwent biventricular repair and achieved good long-term survival and clinical status. The remaining 83 cases underwent staged single-ventricle palliative surgery with 10 early and 7 late deaths.

Many studies suggested that TAPVC, especially obstructed, and/or pulmonary vein stenosis is a risk factor for mortality and long-term survival in children with atrial isomerism (15, 16). Morales et al. reported the outcomes of isomerism patients with TAPVC had improved, and isomerism and non-isomerism survival curves were not significantly different (16). However, they also found that patients with isomerism had significantly higher rates of reoperation due to pulmonary vein stenosis than non-isomerism patients. Foerster et al. reported the outcomes of isomerism with TAPVC (17). Of their 102 study patients (46 with asplenia phenotype, 56 with polysplenia phenotype), 48 (47%) died at a median age of 0.6 months. Sixteen (15.7%) developed pulmonary vein stenosis at a median age of 2 months, with only five (31%) alive at a median of 12.8-years follow-up. The author concluded that pulmonary vein stenosis is common after repair of TAPVC in patients with atrial isomerism and is associated with poor outcomes. In our present study, multiple Cox regression analysis also showed extracardiac TAPVC (especially obstructed) was an independent risk factor for mortality. The risk of residual or recurrent obstruction after extracardiac TAPVC repair was high and consequentially associated with late death.

A large proportion of isomerism patients have a common AV valve, and the propensity for these valves to fail and regurgitate has been identified to be a consistently cited risk factor for poor outcomes (13, 18, 19). Therefore, the AVV repair may necessitate a more aggressive surgical indication, although with more complex surgical techniques. Series reports showed that two-thirds of isomerism patients with AVV regurgitation can achieve successful AVV repair, and successful AVV repair was associated with improved survival, whereas the risk of reintervention is still high (20–22). For isomerism patients, AVV repair often faces greater technical difficulties, and replacement may be the last choice. Our studies (11) and other reports (23) have previously demonstrated that for infants younger than 2-years of age, the mortality of AVV replacement is high. Therefore, the timing and indication for AVV replacement should be determined with caution. In our center, if the patient presented AVV regurgitation that is no more than moderate, the AVV repair was undertaken concomitant with the first palliative surgery, Glenn or Fontan procedure. However, if the patient showed severe AVV regurgitation, the AVV repair or replacement was done alone. In the present cohort, eight patients underwent 9 times reintervention, of which 6 times reintervention occurred in the interval period of Glenn and Fontan procedures. In all 17 deaths, 5 (29.4%) cases were directly related to AVV regurgitation and heart failure. Close follow-up and aggressive intervention are required to avoid further impairment of cardiac function and pulmonary vessels.

In 2016, a case–control study showed that patients with atrial isomerism still face a significantly higher hospital mortality rate in first-stage palliative surgery compared to patients without isomerism, whereas there was no significant difference in interstage mortality or survival after the Glenn and Fontan procedures (2). In our present cohort of patients, 21 cases (25.3%) required first palliative surgery, including 11 of modified B-T shunt, 8 of TAPVC repair, and 7 of PAB. Five patients suffered from early death with a mortality of 23.8%. There were four late deaths, resulting in a total mortality of 42.9%. Multivariate Cox regression showed that the requirement for first-stage palliative surgery was an independent risk factor for mortality. There were fewer neonatal cases in the present cohort, and it was not found to be associated with mortality.

The Fontan completion rate in the present cohort was 43.4%, which was significantly lower than that in patients without isomerism during the same period at our center (43.4% vs. 59.0%, *p* = 0.001). This was consistent with previous reports (24). Despite older age at Fontan procedure (median age: 48 months, range: 26.6–161.3 months), the 1-, 5-, and 8-year survival after Fontan procedure were 94.4, 87.4, and 80.7%, respectively. There was no significant difference compared to patients without isomerism (10). This result indicated that once Fontan circulation is successfully completed, it does not appear to be an important risk factor for its failure, which was similar to Marathe's latest report (5). We also found that the difference of 5-year survival between RAI and LAI was also not statistically significant (*p* = 0.506).

### Limitations

Our study has several limitations (1). This is a retrospective study with the inherent limitations of such studies. Propensity score matching may help to overcome this limitation (2). Our results are limited by the small number of patients with left atrial isomerism. The limited patient numbers and short follow-up time may contribute less sensitivity and may not detect differences that may exist should a larger cohort with longer follow-up be studied (3). This is a single-centered study. Our results do not represent the overall practice and outcomes in China as there is large heterogeneity of patient populations (4). The 13-year study period represents our developing course and the follow-up and surgical strategies and management that is still being refined.

## Conclusion

Despite even greater clinical diversity, the surgical outcomes of atrial isomerism with cardiac malformation are improving in China, and early and intermediate outcomes are comparable to previously reported cohorts. Concomitant extracardiac TAPVC, moderate or severe AVV regurgitation, and the requirement for a first-stage palliative surgery are still risk factors for mortality. Refinements and improvements in perioperative and follow-up management are warranted to further improve the clinical outcomes of this particularly complex group of patients.

## Data Availability Statement

The original contributions presented in the study are included in the article/supplementary material, further inquiries can be directed to the corresponding author/s.

## Ethics Statement

The studies involving human participants were reviewed and approved by Guangzhou Women and Children's Medical Center. Written informed consent to participate in this study was provided by the participants' legal guardian/next of kin. Written informed consent was obtained from the individual(s), and minor(s)' legal guardian/next of kin, for the publication of any potentially identifiable images or data included in this article.

## Author Contributions

M-HZ and FC: conceptualization, data curation, investigation, and writing—review and editing. LM: conceptualization, data curation, and writing—review and editing. W-DC: data curation and formal analysis. W-LL: writing—original draft. JL: conceptualization and writing—review and editing. X-XC: conceptualization, investigation, and supervision. All authors contributed to the article and approved the submitted version.

## Funding

This study was supported by the Key-Area Research and Development Program of Guangdong Province (2019B020227001) and High-Level Clinical Key Subjects Construction Project of Guangzhou City (011102-02).

## Conflict of Interest

The authors declare that the research was conducted in the absence of any commercial or financial relationships that could be construed as a potential conflict of interest.

## Publisher's Note

All claims expressed in this article are solely those of the authors and do not necessarily represent those of their affiliated organizations, or those of the publisher, the editors and the reviewers. Any product that may be evaluated in this article, or claim that may be made by its manufacturer, is not guaranteed or endorsed by the publisher.
